# Roles of Energy/Charge Cascades and Intermixed Layers at Donor/Acceptor Interfaces in Organic Solar Cells

**DOI:** 10.1038/srep29529

**Published:** 2016-07-12

**Authors:** Kyohei Nakano, Kaori Suzuki, Yujiao Chen, Keisuke Tajima

**Affiliations:** 1 RIKEN Center for Emergent Matter Science (CEMS), 2-1 Hirosawa, Wako, Saitama 351-0198, Japan; 2 Precursory Research for Embryonic Science and Technology (PRESTO), Japan Science and Technology Agency, 4-1-8 Honcho, Kawaguchi, Saitama 332-0012, Japan

## Abstract

The secret to the success of mixed bulk heterojunctions (BHJs) in yielding highly efficient organic solar cells (OSCs) could reside in the molecular structures at their donor/acceptor (D/A) interfaces. In this study, we aimed to determine the effects of energy and charge cascade structures at the interfaces by using well-defined planar heterojunctions (PHJs) as a model system. The results showed that (1) the charge cascade structure enhanced *V*_OC_ because it shuts down the recombination pathway through charge transfer (CT) state with a low energy, (2) the charge cascade layer having a wider energy gap than the bulk material decreased *J*_SC_ because the diffusion of the excitons from the bulk to D/A interface was blocked; the energy of the cascade layers must be appropriately arranged for both the charges and the excitons, and (3) molecular intermixing in the cascade layer opened the recombination path through the low-energy CT state and decreased *V*_OC_. Based on these findings, we propose improved structures for D/A interfaces in BHJs.

Recent years have seen the development of solution-processed organic solar cells (OSCs) based on mixed bulk-heterojunction (BHJ) structures that have yielded power conversion efficiency of over 10% with high external quantum efficiency (EQE) values of over 80%^1^,^2^,^3^. This development is truly remarkable considering the simplicity of the method to prepare the organic films used in these cells: casting a solution containing electron-donating and electron-accepting organic semiconducting materials, such as π-conjugated polymers and fullerene derivatives, respectively. A proposal to explain the high performance of the mixed BHJ structures has been put forth. In brief, according to the results of various analytical methods, the donor and acceptor molecules generally form relatively pure phases, separated on the nanoscale by a mixture of these molecules^4^,^5^. Since the structures in the intermixed domains tend to be less ordered (both with regards to molecular conformation and the state of aggregation), their energy gap is often wider than that of the pristine material. This difference in turn leads to a difference between the energy levels of the interface and pure domains, effectively promoting the transport of photogenerated geminate charge pairs from the interface to the pure domains^6^,^7^. It was hypothesized that this “cascade” energy structure at the donor/acceptor (D/A) interfaces induced by the interfacial disorder could be the driving force to overcome the strong Coulomb interaction of the charge pairs in organic materials with low dielectric constants.

To understand how the very high photocurrent generation observed in the mixed BHJ has been obtained, however, one must also consider the efficient flow of the photogenerated excitons to the charge separation interfaces between the domains^8^,^9^,^10^. From this viewpoint, there are three kinds of interfacial cascades: those involving charge transfer (i.e., a “charge cascade”), energy transfer (“energy cascade”) or both, as shown schematically in Fig. 1. They have different effects on the performances of the OSCs and should be discussed separately, although both are often simply called “cascade” in many papers. More accurately, the phase-separated structures with the intermixed domains proposed for the mixed BHJ make use of the charge cascade (possibly of both holes and electrons) but not the energy cascade since the energy gradient opposes the favorable direction if the mixed domains have larger optical gaps than the pristine domains (Fig. 1a). This could be a disadvantage of the mixed BHJ for the collection of exciton energy. Also note that the effects of the energy cascades on the recombination processes in the mixed BHJ are relatively unclear, since little is known about at which interfaces of the three domains charge generation and recombination mainly occur. Where these phenomena occur should be closely related to the short-circuit current density (*J*_SC_) and open-circuit voltage (*V*_OC_) of the OSCs. Therefore, it is important to understand the functions of the mixed interlayer in the BHJ in order to improve the OSC performance.

Well-defined energy and charge cascade structures at the donor/acceptor interfaces have been constructed in planar heterojunctions (PHJs)^11^,^12^,^13^,^14^,^15^,^16^. These studies have been motivated by interest in either using PHJs as models of the interfaces in mixed BHJs, or pursuing the high efficiency of PHJ-type OSCs. It has been reported that the energy cascade toward the charge-generation interface helps the interface collect more excitons^10^. A high efficiency of 8.4% was achieved by optimizing the multilayered structures for the energy cascade^17^. In this case, the interlayer was made relatively thick, in the range of 10–20 nm at the optimum conditions, reflecting the long-range nature of the energy transfer in crystalline molecular semiconductors. On the other hand, it was demonstrated that a charge cascade can facilitate charge generation and prevent recombination if the interlayer is very thin (1–3 nm)^18^,^19^,^20^. Yet, how each of the charge and energy cascade structures simultaneously affects the charge generation and loss processes, such as exciton collection, and geminate and bimolecular recombination is not fully understood. Moreover, the effect of the interlayers containing mixtures of molecules at the D/A interface on these factors is still unclear, although an experimental approach has been recently taken for preparing molecular OSCs by vacuum deposition^21^.

In this study, we investigated the effects of various interlayers in PHJ OSCs fabricated by using the contact film transfer (CFT) technique. We first determined the requirements for each of the energy and charge cascade layers to achieve efficient charge generation by using an interlayer of a pure material (Fig. 2a). Next, we showed that the intermixing in the interlayers largely affected the interface determining the charge-transfer state (CT state) (Fig. 2b). Based on these results, the functions of the intermixed layers at the D/A interfaces are discussed, and the discussion is then extended to intermixed layers in the mixed BHJ.

## Results

Regioregular poly(3-hexylthiophene-2,5-diyl) (P3HT) and [6,6]-phenyl C_61_ butyric acid methyl ester (PCBM) were used as the bulk donor and acceptor layers, respectively. We fabricated solar cell devices using methods similar to those in our previous reports^19^,^22^,^23^,^24^. A PCBM acceptor was spin-coated onto an electrode/electron transport layer (indium tin oxide [ITO]/ZnO), and the interlayer and P3HT films were successively transferred onto the PCBM layer by CFT. MoO_x_ and Ag were then thermally evaporated onto the planar heterojunction structure as the hole transport layer and back electrode, respectively. The device structure was ITO/ZnO/PCBM (28 nm)//interlayer//P3HT (47 nm)/MoO_x_ (7.5 nm)/Ag (70 nm), where //denotes the interface created by the CFT method. The thickness of the layers other than the interlayer was fixed to eliminate the effects of differences in the optical absorption and charge transport in the bulk layers.

**Figure 1:**
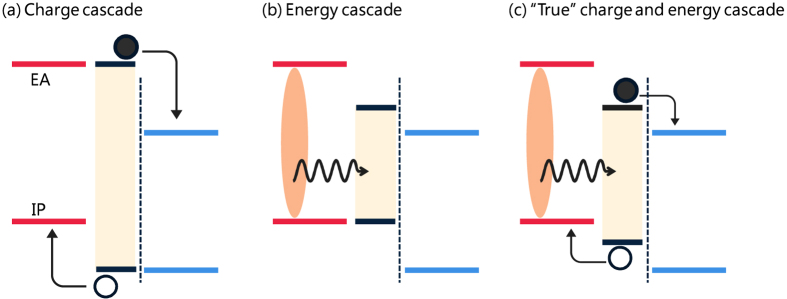
Schematic energy diagrams with cascades at donor/acceptor interfaces involving (**a**) charge (hole) transfer, (**b**) energy transfer and (**c**) both types of transfer. Closed and open circles represent electrons and holes, respectively, and the wavy arrows and dashed lines indicate the energy transfer and the donor/acceptor interfaces, respectively.

To investigate the requirements of the energy cascade for efficient charge generation, we used two polymers for the interlayer: regiorandom poly(3-hexylthiophene-2,5-diyl) (ran-P3HT) and poly({4,8-bis[(2-ethylhexyl)oxy]benzo[1,2-*b*:4,5-b′]dithiophene-2,6-diyl}{3-fluoro-2-[(2-ethylhexyl)oxycarbonyl]thieno[3,4-*b*]thiophenediyl}) (PTB7). Compared to P3HT, ran-P3HT has a wider optical energy gap due to its less ordered structure in the films, while PTB7 has a narrower gap (see Fig. 3)^25^,^26^,^27^. Therefore, the interlayer of ran-P3HT could produce only the charge (hole) cascade (Fig. 1a), while the interlayer of PTB7 could produce both charge and energy cascades (Fig. 1c). The multilayer structures produced were PCBM//PTB7//P3HT and PCBM//ran-P3HT//P3HT with various interlayer thicknesses (1.3–11 nm). The thicknesses of the interlayers were determined by measuring the absorbance values of the films before the transfer process; calibration was performed using linear relationship between the thickness measured by X-ray reflectivity (XRR) and the absorbance (see Figures S1 and S3 in the Supporting information). We confirmed by atomic force microscopy that the surface of each film before and after the transfer was very smooth (root-mean-square roughness <0.6 nm) (Figures S2 and S4). According to XRR fits (Figure S5 and Table S1), there was no detectable mixing of the materials at the interfaces after the transfer process. This lack of mixing was apparently due to the film transfer having been conducted under mild conditions, that is, at room temperature and with neither pressure nor heat applied.

The *J*_SC_ and *V*_OC_ values of the PHJ OSCs with various interlayer thicknesses under irradiation of AM1.5 100 mW/cm^2^ are shown in Fig. 4a,b (the current density-voltage (*J-V*) curves are shown in Figure S6, and the extracted device parameters are listed in Tables S2 and S3). The insertion of a ran-P3HT interlayer with a thickness of 1.3 nm caused a decrease of the *J*_SC_ from 1.1 to 0.67 mA/cm^2^; as the thickness was increased, *J*_SC_ slightly recovered. In contrast, the insertion of PTB7 interlayers with thicknesses of 1.4 nm and 2.7 nm increased *J*_SC_ to 1.5 mA/cm^2^ and 2.2 mA/cm^2^, respectively; *J*_SC_ remained at about 2.2 mA/cm^2^ as the thickness was increased further.

EQE spectra of the OSCs with ran-P3HT interlayers of various thicknesses are shown in Fig. 4c. The photoresponse at about 620 nm observed without the interlayer was from the absorption of P3HT (the absorption spectra are shown in Figure S7), while the response at around 420 nm was mainly from PCBM. The response at 620 nm almost disappeared in the presence of a ran-P3HT interlayer with a thickness of 1.3 nm or greater, while there was little change in the other peak. This result indicates that the photocurrent generation from excitons in the P3HT layer was shut down because the ran-P3HT interlayer prevented the excitons from diffusing to the PCBM interface. Note that the diffusion of excitons was entirely blocked even with only a 1.3-nm-thick ran-P3HT layer, and this result suggests that the tunneling charge transfer from P3HT to PCBM through ran-P3HT layer was negligible in our set up^6^. In contrast, inclusion of the PTB7 interlayer slightly increased the photoresponse at 620 nm and yielded a new photoresponse at 680 nm that arose from the absorption of PTB7 (Fig. 4d). Taking the overlap of the photoluminescence of P3HT and the light absorption of PTB7 and ran-P3HT films into consideration (Figure S7b), we conclude that Förster energy transfer was allowed from P3HT to PTB7 owing to the narrow energy gap of PTB7, so the excitons generated in both P3HT and PTB7 were able to contribute to the photocurrent. The enhancement of EQE at around 420 nm may be attributed to weak absorption of PTB7, additional Förster energy transfer from PCBM to PTB7, or improved charge generation efficiency by the cascade layer, but it is difficult to discuss these contributions separately. As the thickness of the interlayer of PTB7 was increased (to ~10 nm), the photoresponse from P3HT stopped being apparent in the EQE spectrum, while the contribution from PTB7 slightly increased. This result suggests that increasing the thickness of the layers involved in the energy cascade beyond the exciton diffusion length of the semiconducting polymer (<10 nm) diminished the advantage of the exciton collection by Förster energy transfer.

To investigate differences in the charge generation process, the temperature dependence of *J*_SC_ was measured. Table S4 summarizes the activation energy of *J*_SC_ (*∆*) determined by the temperature dependence measurements (The Arrhenius plots from which these *∆* values were derived are shown in Figure S8). The value of *∆* for the PCBM//P3HT bilayer OSC was very low (10.4 meV), less than half of the energy of room temperature (26 meV at 300 K) and similar to the value recently reported for a BHJ OSC of P3HT:PCBM (9 meV)^28^. When ran-P3HT was inserted, *∆* values slightly increased to 11.7–15.8 meV, which is still smaller than the energy of room temperature, although CT state dissociation efficiency in ran-P3TH:PCBM was reported to be much lower (~0.3) than in P3HT:PCBM (~0.9)^29^. On the other hand, OSCs with the thin (1.4 and 2.7 nm) interlayers of PTB7 showed small *∆* values (9.3–10.5 meV) comparable to those for PCBM//P3HT interface; however as the thickness of the PTB7 interlayer was increased, the *∆* values became larger (up to 26.4 meV) and comparable to the thermal energy of room temperature. Light intensity dependence of *J*_SC_ indicates that the bimolecular recombination does not have a large influence on these devices at the short circuit condition even at the low temperature (Figure S17), as often observed for PHJs and the mixed BHJs with optimized morphology^30^,^31^,^32^. Temperature dependence of the charge transport process and the collection at the electrodes should not be the origin of the observed changes in *∆* since the bulk layers are the same among the devices under comparison. Therefore, the temperature dependence of *J*_SC_ could be attributed to the charge generation process. The smaller *∆* with the thin PTB7 interlayer may be due to efficient charge transfer from the cascade layer to the bulk donor layer. Thick cascade layers may not suppress geminate recombination as predicted by the model^20^. These results show the possibility that the thin charge cascade layers could work to suppress the geminate recombination.

Figure 4b shows the dependence of *V*_OC_ of the devices on the thickness of the pure interlayers. For both P3HT and PTB7, *V*_OC_ gradually increased as the thickness of the interlayer was increased to about 4 nm and then plateaued as the thickness was increased further. This profile can be mainly attributed to the change in the energy of the CT state through which bimolecular recombination occurs in the open-circuit condition. There are two possible CT states involving the interlayers: one with charge pairs at interlayer/PCBM interface and one with charge pairs at the P3HT/PCBM interface through the interlayer. Both of these CT states can have a higher *E*_CT_ than that of P3HT/PCBM (which does not have an interlayer), due to the deeper ionization potential of the interlayer (donor) or the longer distances between the charge pairs. The gradual change of *V*_OC_ with the change in the thickness of the interlayer suggests that the relative contributions of each CT state to bimolecular recombination might depend on the thickness of the interlayer. We recently showed by impedance spectroscopy that the charge cascade structure can cause the depletion of the charges at the interface in the open-circuit condition, so that holes could accumulate in P3HT at the interface with the interlayer^33^. Therefore, with a relatively thin interlayer (<4 nm), a large population of holes in P3HT could still contribute to charge recombination, resulting in the gradual change of *V*_OC_. The *V*_OC_ values with ran-P3HT or PTB7 interlayer converged to the values similar to those of the ran-P3HT//PCBM (0.73 ± 0.02 V) or PTB7//PCBM (0.75 ± 0.01 V) bilayer systems, respectively, for interlayer thicknesses greater than 5 nm because the recombination interface switched completely to the interlayer/PCBM interfaces. A continuous shift of the electroluminescence peaks from the CT state^34^ to higher energy region was observed as the thickness of the ran-P3HT layer was increased (Figure S9), which is consistent with a gradual change of the average CT state energy. There could be small contributions to the changes of *V*_OC_ from the change in the charge generation/recombination dynamics, which requires further investigation by transient optical and electric measurements.

Next, to investigate the effects of D/A intermixing in the interlayer, thermal annealing at 150 °C for 10 min was carried out after the transfer of the interlayers onto the PCBM film. (The detailed procedure is described in the *Methods* section). PCBM was deliberately diffused into the ran-P3HT and PTB7 layers by thermal annealing prior to the transfer of P3HT. Note that we did not use regioregular P3HT for the diffusion experiments because the highly crystalline P3HT probably gives BHJ-like domain structures with the diffused PCBM in the interlayer, which would complicate the interpretation of the results. Since molecularly mixed interlayers are necessary to simulate the intermixed domains in BHJs, and ran-P3HT is known to have a high miscibility with PCBM, we used ran-P3HT for the experiments. The diffusion of PCBM into polymer layers by thermal annealing has been reported by many groups^35^,^36^,^37^. XRR measurements before and after annealing process showed that the density and thickness of the interlayer increased and the thickness of the PCBM layer decreased (Figures S10 and S11 and Tables S7 and S8). These observations are clear evidence for the diffusion of the PCBM into the interlayers. However, the change in density was larger for the ran-P3HT layer than for the PTB7 layer, suggesting a difference in the degree of the PCBM diffusion. X-ray photoelectron spectroscopy of the surfaces of the films also indicated that the ability of PCBM to diffuse into the polymers may differ between ran-P3HT and PTB7; the concentration of sulfur on the surface of ran-P3HT decreased markedly after annealing, even with the thicker interlayer, indicating that the PCBM freely diffused to and saturated the surfaces of the films (Figure S12). On the other hand, the sulfur concentration on the surface decreased only slightly after annealing in the case of PTB7, indicating that less PCBM diffused to the surface. This difference could be related to the different miscibilities of weakly crystalline PTB7 and amorphous ran-P3HT, which could affect the OSC performance (see below).

The performance of the OSCs containing the ran-P3HT:PCBM mixed interlayers is shown in Fig. 5. (The corresponding *J-V* curves and the extracted device parameters are shown in Figure S13 and Table S9, respectively). A photoresponse at about 620 nm from P3HT was observed even for thicknesses of the intermixed interlayers up to 10 nm (Fig. 5c). This observation is in sharp contrast to the case of the pure ran-P3HT interlayer, where the response of the film, even one with a thickness of only 1.3 nm, was almost completely shut down (Fig. 4c). These observations indicate that the excitons generated in the P3HT layer can reach PCBM and dissociate. As described above, PCBM diffused to the surface of the ran-P3HT layer after annealing, so there could be close contact with P3HT. Therefore, the devices with mixed interlayers have two charge separation interfaces, at ran-P3HT/PCBM and P3HT/(diffused) PCBM.

On the other hand, the enhancement of *V*_OC_ provided by the mixed interlayer (Fig. 5b) was not as large as that provided by the pure ran-P3HT interlayer. Even when including the mixed interlayer with a thickness of 10 nm, *V*_OC_ increased from 0.45 V to only 0.53 V, which is much less than the 0.74 V value for the device with the pure 10-nm-thick ran-P3HT interlayer and also less than the 0.60 V value for the device containing the 1.3-nm-thick ran-P3HT interlayer. These observations could be explained as follows. The holes in the P3HT layer can form the CT state with PCBM in the mixed interlayer diffusing to the surface, which has lower energy than that of the CT state of ran-P3HT:PCBM. Consequently, *V*_OC_ was not as high as with the pure ran-P3HT interlayer. In other words, leakage of the free charge carriers through the CT state with lower energy could limit *V*_OC_ in the presence of the mixed interlayer. The electroluminescence of the devices after the PCBM diffusion showed the shift of the CT state emission to a lower energy (peak top from 1.25 eV to 1.1 eV, Figure S18), which further supports the change of the CT state energy after the intermixing.

Figure 6 shows the performance of OSCs with mixed interlayers of PTB7:PCBM. (The *J-V* curves and the extracted device parameters are shown in Figure S14 and Table S10, respectively). Here, *J*_SC_ increased as the thickness of the mixed interlayer was increased, similar to the case of the pure interlayer, but in contrast no plateau was observed for interlayer thicknesses greater than 5.4 nm. According to the EQE values shown in Fig. 6c, this large *J*_SC_ enhancement mainly originated from the additional light absorption of PTB7, though the contribution of P3HT at 620 nm was still observed up to an interlayer thickness of 2.7 nm. These observations suggest that charge generation with the pure interlayer and that with the mixed interlayer were similarly efficient when these interlayers were thin since the excitons in P3HT were able to contribute in both cases through the energy transfer. As the interlayer became thicker, the exciton diffusion in the pure PTB7 interlayer started to limit the contribution of P3HT as discussed above while the photoresponse from PTB7 in the mixed interlayer started to dominate the photocurrent.

**Figure 2:**
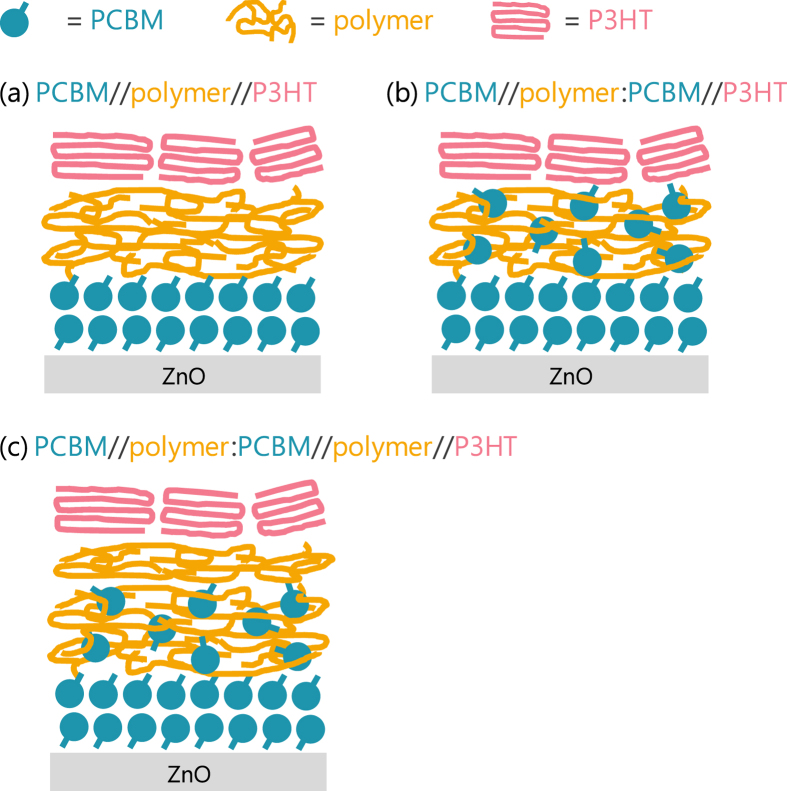
Schematic illustration of PCBM/P3HT PHJ structures with (**a**) pure, (**b**) mixed and (**c**) incompletely mixed interlayers.

**Figure 3:**
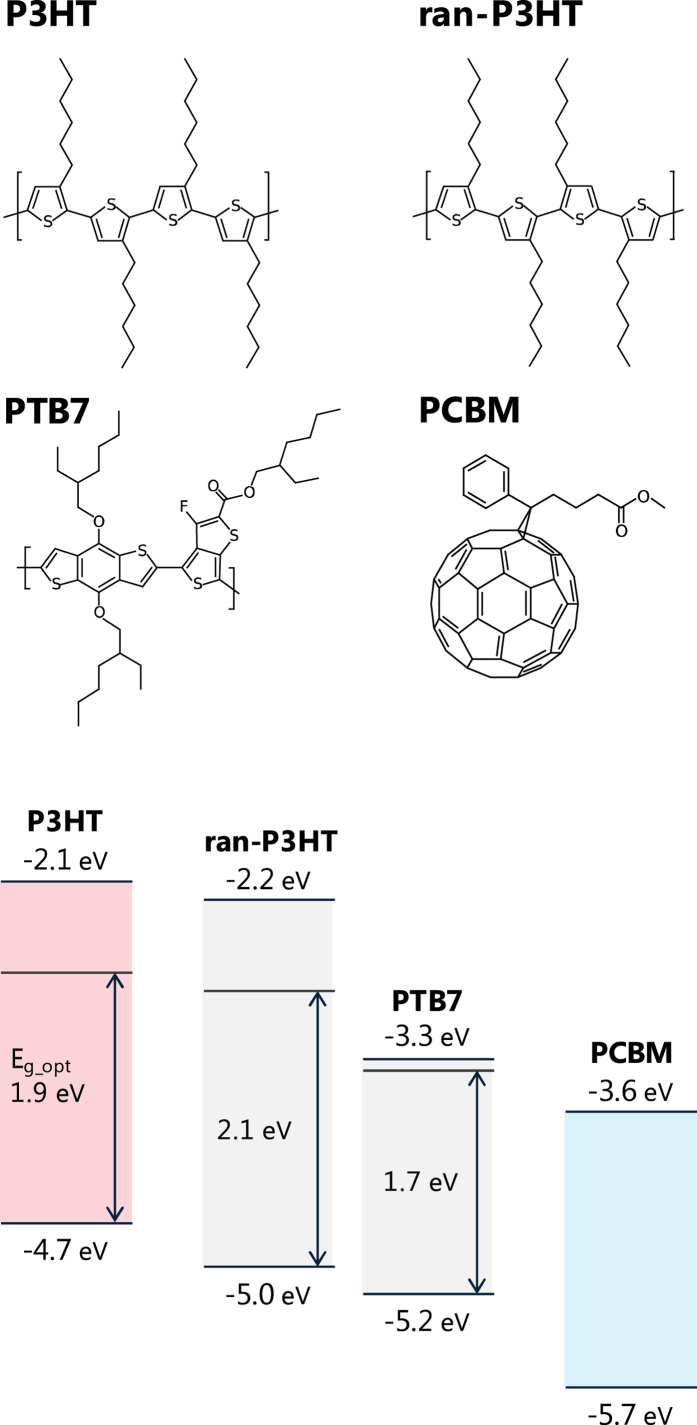
Chemical structures, energy levels and optical energy gaps of the materials used in this study. Energy levels of P3HT, ran-P3HT and PCBM measured with photoemission spectroscopy were taken from refs 25,26. The energy levels of PTB7 measured by cyclic voltammetry is taken from ref. 27. Optical energy gaps (*E*_g_opt_) were determined from the onsets of absorption in the absorption spectra.

**Figure 4:**
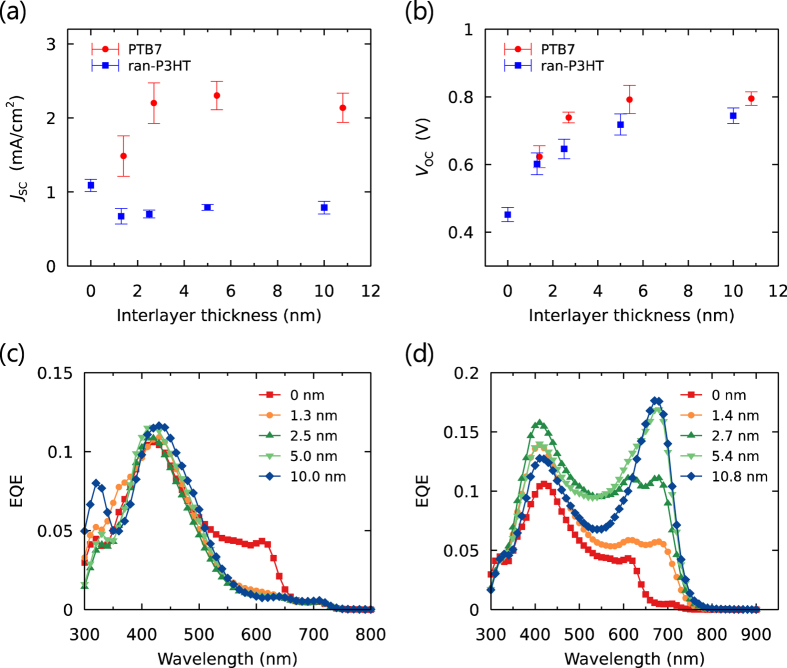
Interlayer thickness dependence of (**a**) the short-circuit current density (*J*_SC_) and (**b**) open-circuit voltage (*V*_OC_) of OSCs under irradiation of AM1.5 simulated sunlight (100 mW/cm^2^). The error bars are the standard deviations calculated from at least 6 devices. EQE plots of the OSCs with (**c**) PCBM//ran-P3HT//P3HT and (**d**) PCBM//PTB7//P3HT structures with various interlayer thicknesses.

**Figure 5:**
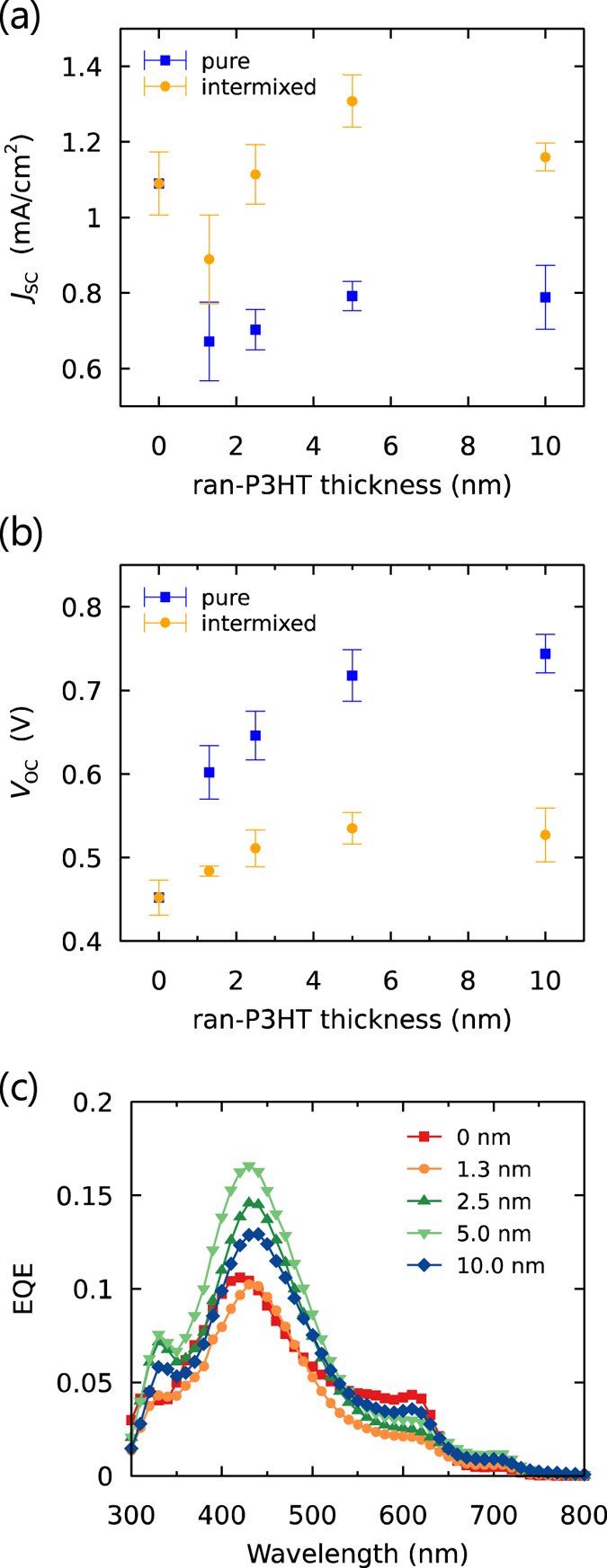
Interlayer thickness dependence of (**a**) short-circuit current density (*J*_SC_) and (**b**) open-circuit voltage (*V*_OC_) for PHJ OSCs with PCBM/ran-P3HT:PCBM//P3HT structures under the irradiation of AM1.5 simulated sunlight (100 mW/cm^2^). The error bars are the standard deviations calculated from at least 6 devices. (**c**) External quantum efficiency (EQE) plots for various interlayer thicknesses.

**Figure 6:**
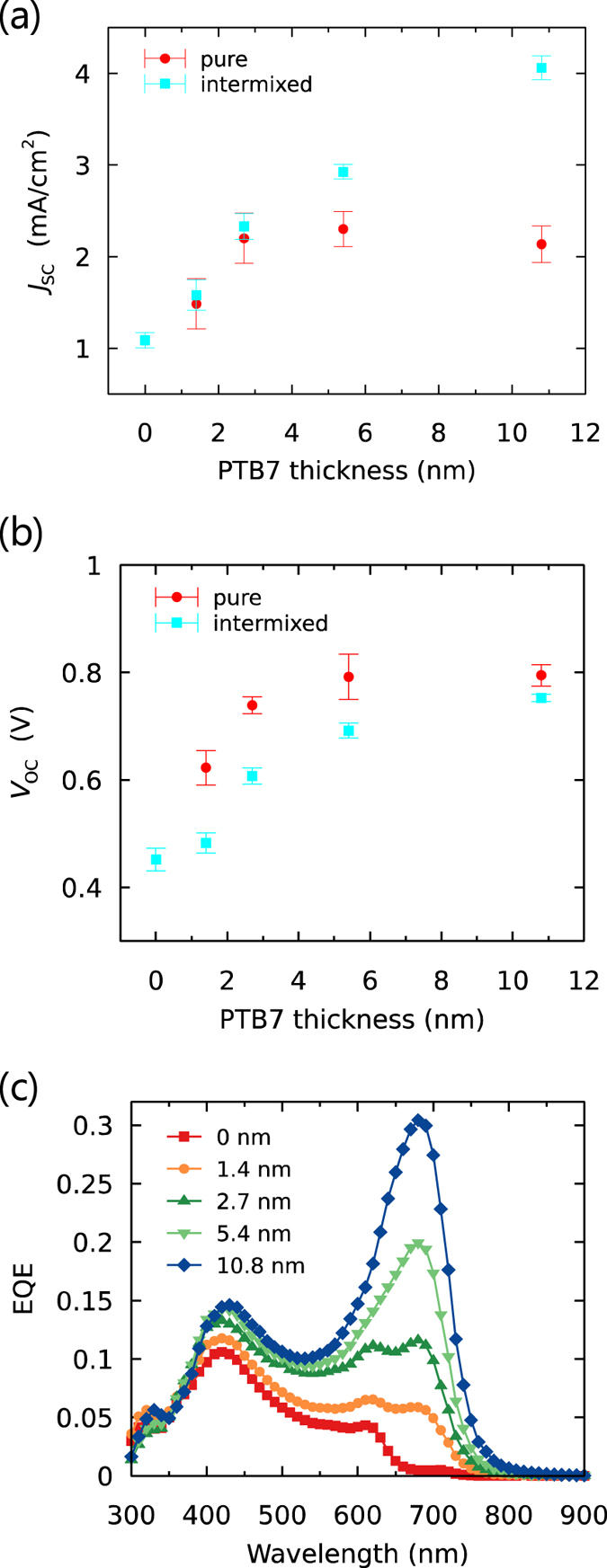
Interlayer thickness dependence of (**a**) short-circuit current density (*J*_SC_) and (**b**) open-circuit voltage (*V*_OC_) for OSCs with PCBM/PTB7:PCBM//P3HT structures under the irradiation of AM1.5 simulated sunlight (100 mW/cm^2^). The error bars are the standard deviations calculated from at least 6 devices. (**c**) EQE plots for various interlayer thicknesses.

Similar to the results for the ran-P3HT:PCBM interlayers, *V*_OC_ was observed to decrease when including the PTB7:PCBM mixed interlayer, but gradually recovered to that for the pure PTB7 interlayer (0.80 V) as the thickness was increased (Fig. 6b). This difference between PTB7:PCBM and ran-P3HT:PCBM could be due to the difference in the distribution of PCBM in the interlayer, as discussed for the results of X-ray photoelectron spectroscopy. A relatively pure layer of PTB7 apparently remained at the interface with P3HT, which suppressed the recombination via a low-energy CT state between P3HT and PCBM (see Fig. 2c for a schematic illustration).

We sought to confirm the loss of the exciton blocking effect and the change in the recombination process by reproducing the structure illustrated in Fig. 2c. For this purpose, an additional CFT process was used to insert a thin (1.3 nm) pure interlayer of ran-P3HT between the 10-nm-thick mixed interlayer and the P3HT layers to form PCBM/ran-P3HT:PCBM//ran-P3HT//P3HT. The insertion of a pure layer onto the intermixed layer shut down the recombination path of P3HT:PCBM: *V*_OC_ increased to 0.72 V, close to the 0.74 V value of OSCs with the pure ran-P3HT interlayer (Table S9). At the same time, this thin pure layer again caused exciton blocking, resulting in the shutdown of the photoresponse from P3HT in the EQE spectra (Figure S15). From these results, we conclude that the mixed interlayers led to charge generation from both the P3HT/PCBM and interlayer/PCBM interfaces, but the recombination path with the lower CT state energy opened at P3HT/PCBM, so that the gain of *V*_OC_ with the pure interlayer was lost.

## Discussion

The results for the pure and the mixed interlayers suggest that an energy cascade is necessary to collect excitons from the bulk layer. Even the thinnest interlayer (1.3 nm) with a relatively wide band gap almost completely blocked the diffusion of excitons. The lower activation energy for charge generation observed for the thin (<2.7 nm) PTB7 interlayers compared with the thicker ones suggests that the charge cascade could suppress geminate recombination. On the other hand, the bimolecular recombination path through the low-energy CT state was apparently suppressed by insertion of the charge cascade interlayer with a thickness of ~4 nm, which was observed to yield a high *V*_OC_. To achieve both efficient exciton collection and suppression of *V*_OC_ loss, a “true” cascade layer with a thickness optimized as it was in PCBM//PTB7//P3HT is necessary. The intermixing in the interlayers compromised these effects and reduced *V*_OC_ since they could provide a recombination pathway with a lower CT state energy. We conclude that a pure and thin true cascade layer is an ideal interfacial structure for simultaneously achieving efficient charge generation and suppressed recombination in PHJs.

It would be interesting to compare the current cascade systems with PCBM/P3HT PHJs having interlayers of the insulating fluoropolymer CYTOP we reported previously^38^. In that case, the EQE results showed that the photoresponses from both P3HT and PCBM decreased monotonically and simultaneously as the CYTOP interlayer was made thicker, which is in contrast to the case of PCBM//ran-P3HT//P3HT where only the responses from P3HT decreased. As a result, the CYTOP interlayer led to a drastic decrease of *J*_SC_. On the other hand, inclusion of the CYTOP interlayer with the optimum thickness (1 nm) increased *V*_OC_ of the OSCs by 0.1 V, which was attributed to the longer distance of the charge pair and thus the higher energy of the CT state. This gain is comparable to those observed for ran-P3HT (+0.15 V) and PTB7 interlayers (+0.17 V) with similar thicknesses (1.3 and 1.4 nm, respectively). A thicker CYTOP layer might have been expected to further increase *V*_OC_, but instead the photocurrent was observed to drop rapidly, which decreased the charge density and *V*_OC_.

Finally, we discuss the implications of our results for the current mixed BHJ systems. For mixed BHJ structures that can be described as phase-separated pure donor and pure acceptor domains with a narrow mixed domain in between, the interfaces would be similar to those in P3HT/ran-P3HT:PCBM/PCBM. According to our results, this structure suffered from a trade-off between the exciton blocking effect by the ran-P3HT domain and the recombination pathway through the relatively low-energy CT state between P3HT and PCBM, and this trade-off depended on how well the materials mixed together in the interlayer. Even if it were possible to selectively replace ran-P3HT in the mixed domain with a lower bandgap material, there would still be a loss of *V*_OC_ if the pure donor domain makes contact with the acceptor. A thin blocking layer with the charge cascade would be necessary to avoid this loss. Alternatively, if we could avoid the use of domains of mixtures of molecules and completely rely on the true cascade interface between the pure donor and pure acceptor domains, that would also be an ideal structure although the optimum domain sizes would be strictly limited by the exciton diffusion length.

The results also imply that it is possible to improve the performance of the ternary blend systems that are currently being studied intensively. Compared to the corresponding binary system, many ternary blend systems have shown increases in *J*_SC_ due to better absorption and energy transfer but with no or only a slight increase in *V*_OC_^39^,^40^. These observations are probably due to the relatively low-energy CT state between the bulk donor and the acceptor limiting *V*_OC_ in these systems because of the low purity in the interlayer of the third material. Ohkita and coworkers recently reported that a P3HT:PCBM BHJ heavily doped with a near-IR-absorbing dye (30 wt%) showed an approximately 50 mV increase of *V*_OC_^41^. This increase could be due to the better coverage of the D/A interface with the dye, which somewhat suppresses the recombination; however, if the recombination path from the low-energy CT state (P3HT/PCBM) is completely shut down, a much larger *V*_OC_ gain can be expected. Assuming that the dyes were located in the mixed phases of disordered P3HT and PCBM as proposed by the authors, the device performance may have still been limited by recombination via the CT state of P3HT/PCBM, similar to the case for our PHJs with the intermixed interlayers.

Therefore, the next important challenge for the development of OSCs is to realize cascade structures in BHJs with high purity of the material in the domains. For this purpose, more precise placement of the materials at the nanoscale in the organic thin films is necessary, which might be beyond the capacity for mixed BHJs. Instead, such an improvement may be realized by self-organization of single materials based on molecular designs such as the microphase separation of semiconducting block copolymers^42^,^43^,^44^,^45^.

## Methods

### Materials

ran-P3HT (*M*_w_: 60,000–95,000; Rieke Metals), regioregular P3HT (lisicon SP001, Merck), PTB7 (*M*_w_: 128,000; 1-Material) and PCBM (purity: 99.5%; Solenne) were purchased from commercial suppliers and used as received.

### Device fabrication

For each device, a patterned ITO-coated glass substrate was cleaned by sequential ultrasonication in detergent solution, water, 2-propanol and acetone, followed by UV-O_3_ treatment. The ZnO layer was prepared by applying the sol-gel method as follows: a mass of 100 mg of zinc acetate dihydrate was dissolved into 1.6 ml ethanol. 2-Aminoethanol (28.4 μl) was added to this solution in order to stabilize the precursor. The resulting solution was spin-coated onto ITO at 3000 rpm for 30 s. After spin-coating, the resulting IT/ZnO substrate was annealed at 200 °C 30 min in air. The ZnO layer (with a thickness of about 30 nm) was cleaned by sonification using 2-propanol and acetone. A 10 mg/ml chlorobenzene solution of PCBM was spin-coated onto each of the ITO/ZnO substrates at 600 rpm for 60 s to give a 28-nm-thick film. The substrates were thermally annealed at 150 °C for 5 min inside a N_2_-filled glovebox to crystallize PCBM, which prevents any intermixing of PCBM with the interlayer^46^. To induce the diffusion of PCBM into the interlayers, thermal annealing was carried out after the transfer of the interlayer (150 °C for 10 min). Photographic instruction of each step for the contact film transfer method is shown in supplementary information (Figure S16). Aqueous poly(sodium 4-styrenesulfonate) (PSS; 30 mg/ml; *M*_w_: 70,000; Aldrich) was spin-coated onto a pre-cleaned glass substrate at 3000 rpm for 30 s (RMS roughness of the surface: 0.3 nm). A solution of P3HT (10 mg/ml) in chlorobenzene was spin-coated onto glass/PSS substrates at 1000 rpm for 60 s, resulting in a P3HT thickness of 47 nm. Chlorobenzene solutions of various concentrations of ran-P3HT or PTB7 were also spin-coated onto respective PSS layers. The glass/PSS/polymer substrate was gently placed upside down on the ITO/ZnO/PCBM substrate, and one drop of water was placed on the edge of the two substrates. Water selectively penetrated into and dissolved the PSS layer, allowing the polymer layer to be transferred onto the PCBM layer. A MoO_3_ hole-transporting layer (7.5 nm) and Ag electrodes (70 nm) were deposited by thermal evaporation under high vacuum (~10^−4^ Pa) through a metal mask. All the samples were encapsulated with glass cap and UV-curable resin in a dry N_2_-filled glovebox.

### Measurements

The film thicknesses of PCBM and P3HT were determined by using a surface profiler (DEKTAK 6M, Bruker). A part of each of these thin films was removed by applying chloroform, and the height of the resulting edge was measured. At least six points were measured and averaged. XRR was performed by using a Rigaku Smartlab X-ray diffractometer, and the reflection patterns were fitted by using GlobalFit software (Rigaku). Monochromatized Cu Kα radiation (λ = 0.154 nm) was generated at 45 kV and 200 mA. To conduct XRR measurements of the interlayers, the samples were prepared by using the float-off film transfer method because the hydrophilic SiO_2_ surface was not suitable for the CFT method. In this float-off film transfer method, the thin polymer films on the glass/PSS substrates were floated off onto water, and the films then became adhered to Si/SiO_2_ substrates. The films with a thickness of less than 2 nm could not be found by the naked eye, and thus we could not perform XRR or optical microscopy observation of 1.3-nm-thick ran-P3HT and 1.4-nm-thick PTB7 layers and instead used UV-Vis absorption spectroscopy to measure the thickness (see Supporting information). These absorption spectra were measured with a UV-Vis spectrophotometer (V-670, JASCO) from 350 nm to 900 nm. Atomic force microscopy images were obtained with a 5400 scanning probe microscope (Agilent Technologies) in tapping mode. The *J-V* characteristics of the devices were measured under simulated solar illumination (AM 1.5, 100 mW/cm^2^) from a solar simulator based on a 150 W Xe lamp (PEC-L11, Peccell Technologies). The light intensity was calibrated with a standard silicon solar cell (BS520, Bunkoh-Keiki). The active area of each device was defined by using a 0.12 cm^2^ metal photo mask. The EQE of each device was measured with monochromatic light (SM-250F, Bunkoh-Keiki). The *J-V* characteristics of the devices at different temperatures (r.t. to −60 °C) were measured in a stainless steel chamber with needle probes (KITANO SEIKI) under the irradiation of a white LED light (10 W LED XM-L, Cree). The temperature was measured at the surface of the sample by using sheet-type thermocouples. Electroluminescence was measured by using a spectrofluorometer equipped with a liquid N_2_-cooled InGaAs detector (Nanolog, Horiba), and a constant DC voltage was applied to the devices using a source measurement unit (6243, ADC).

## Additional Information

**How to cite this article**: Nakano, K. *et al*. Roles of Energy/Charge Cascades and Intermixed Layers at Donor/Acceptor Interfaces in Organic Solar Cells. *Sci. Rep.* **6**, 29529; doi: 10.1038/srep29529 (2016).

## Supplementary Material

Supplementary Information
