# Supplementary material for: Roles of Energy/Charge Cascades and Intermixed Layers at Donor/Acceptor Interfaces in Organic Solar Cells
Source: Sci Rep. 2016 Jul 12;6:29529. doi: 10.1038/srep29529 (PMC4941572; doi:10.1038/srep29529)
Supplement: Supplementary Information [file srep29529-s1.pdf]

Supporting information for

Roles of Energy/Charge Cascades and Intermixed Layers at Donor/Acceptor Interfaces in Organic Solar Cells

Kyohei Nakano[1], Kaori Suzuki[1], Yujiao Chen[1] and Keisuke Tajima[1,2,a]

[1]*RIKEN Center for Emergent Matter Science (CEMS), 2-1 Hirosawa, Wako, Saitama 351-0198, Japan,*

[2]*Precursory Research for Embryonic Science and Technology (PRESTO), Japan Science and Technology Agency, 4-1-8 Honcho, Kawaguchi, Saitama 332-0012, Japan*

[a)]Electronic mail: keisuke.tajima@riken.jp

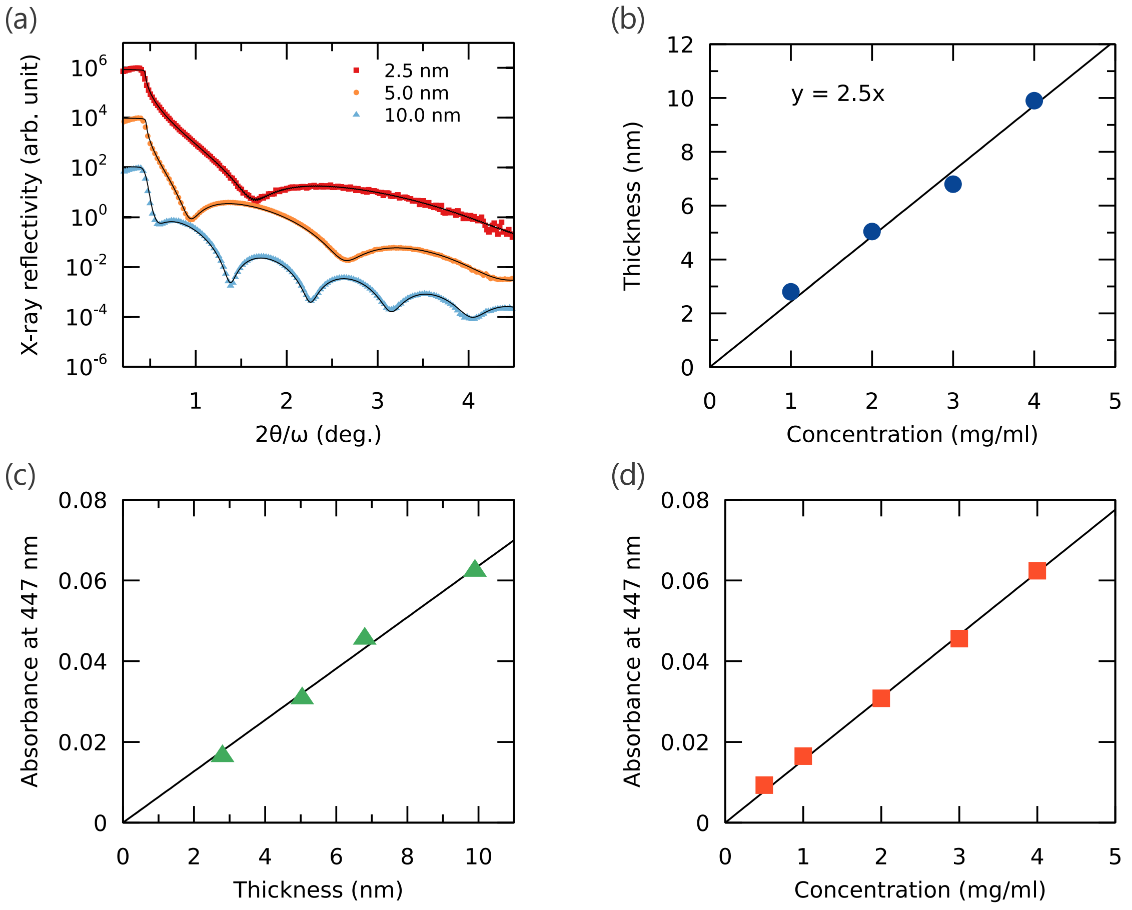

Figure S1. (a) X-ray reflectivity (XRR) of each of three regiorandom poly(3-hexylthiophene-2,5-diyl) (ran-P3HT) thin films (with indicated thicknesses between 2.5 nm and 10.0 nm) transferred onto Si/SiO_2 (300 nm) substrates. The fittings were performed with a multilayer model of Si/SiO_2/ran-P3HT with flat interfaces, and with the thickness and density of ran-P3HT used as the fitting parameters (solid lines). (b) Film thickness (measured by XRR) plotted against concentration of ran-P3HT in chlorobenzene used for spin-coating. (c) Absorbance at 447 nm for the films spin-coated on glass/PSS substrates plotted against the thickness determined by XRR. (d) Absorbance at 447 nm plotted against the concentration. The relationships between these three factors have good linearity in the ranges measured. Thicknesses reported in this study were derived from these linear relationships.

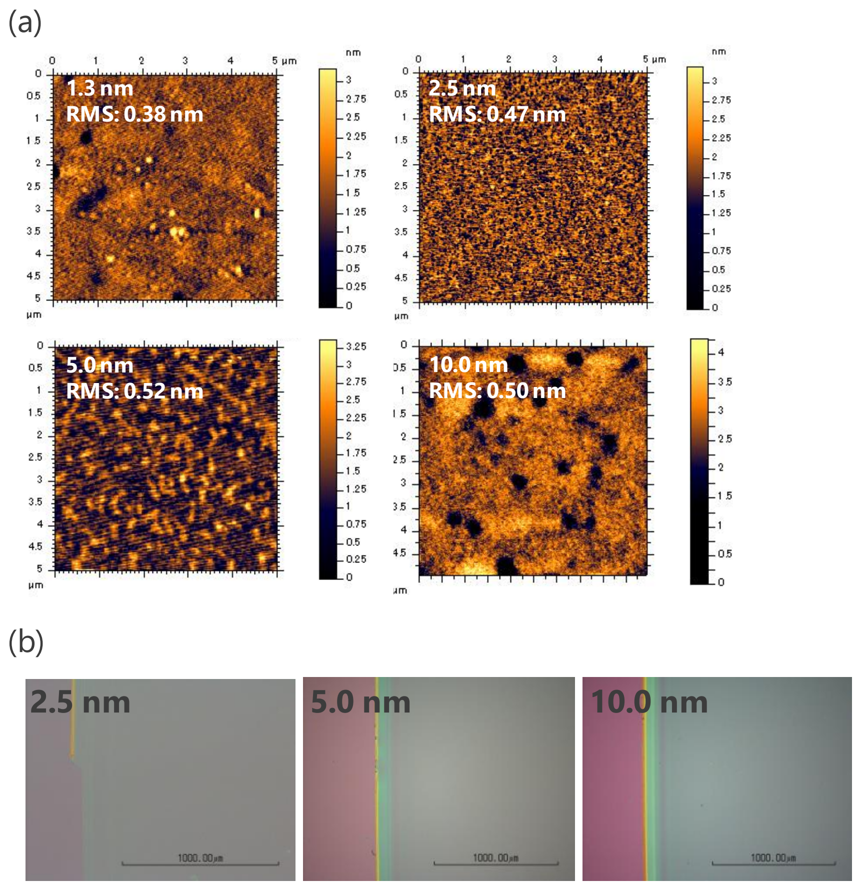

Figure S2. (a) Atomic force microscopy (AFM) height images of the surfaces of the ran-P3HT thin films spin-coated on glass/poly(sodium 4-styrenesulfonate) (PSS) substrates. The film thickness and the root-mean-square (RMS) roughness are presented for each case. (b) Optical microscope images of the ran-P3HT thin films transferred onto the Si/SiO_2 substrate. The left side of each image in (b) is the uncovered surface of the substrate.

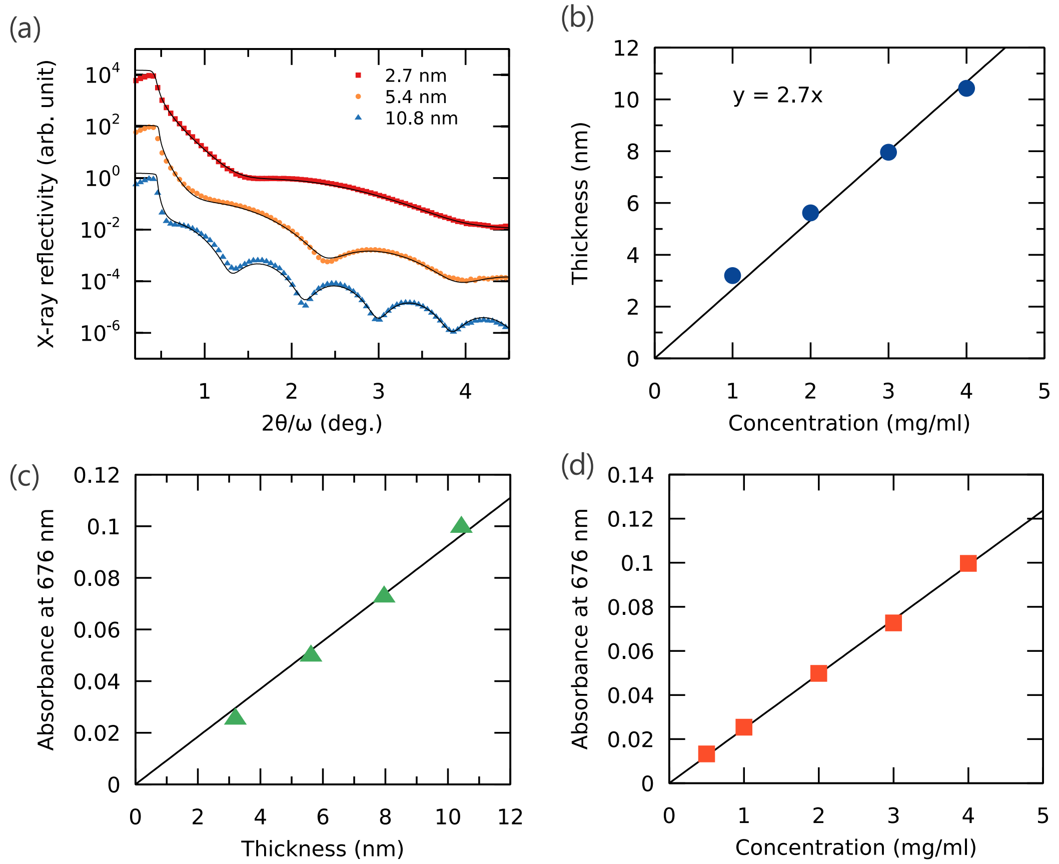

Figure S3. (a) XRR of poly({4,8-bis[(2-ethylhexyl)oxy]benzo[1,2-*b*:4,5-b′]dithiophene-2,6-diyl}{3-fluoro-2-[(2-ethylhexyl)oxycarbonyl]thieno[3,4-*b*]thiophenediyl}) (PTB7) thin films (with indicated thicknesses) transferred onto respective Si/SiO_2 (300 nm) substrates. The fittings were performed with a multilayer model of Si/SiO_2/PTB7 with flat interfaces, and with the thickness and density of PTB7 used as the fitting parameters (solid lines). (b) Film thickness (measured by XRR) plotted against concentration of PTB7 in chlorobenzene used for spin-coating. (c) Absorbance at 676 nm for the films spin-coated on glass/PSS substrates plotted against the thickness determined by XRR. (d) Absorbance at 676 nm plotted against the concentration. The relationships between these three factors have good linearity in the ranges measured. Thicknesses reported in this study were derived from these linear relationships.

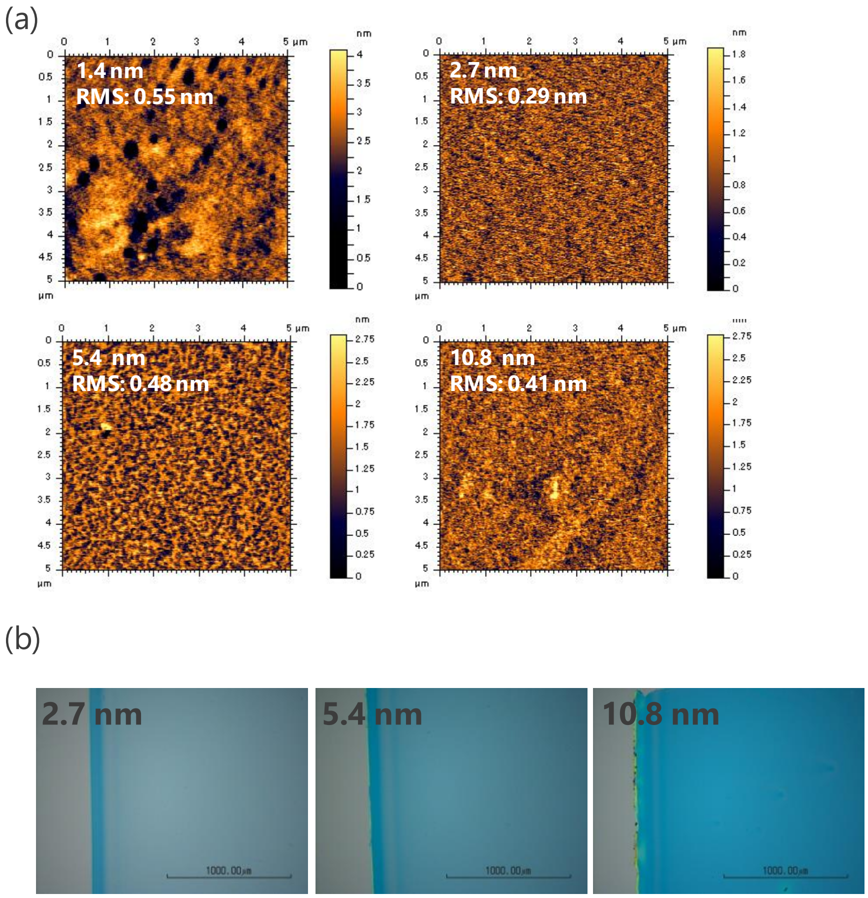

Figure S4. (a) AFM height images of the surfaces of the PTB7 thin films spin coated on glass/PSS substrates. The thickness and RMS roughness are presented for each case. (b) Optical microscope images of the PTB7 thin films transferred onto Si/SiO_2 substrates. The left side of each image in (b) is the uncovered surface of the substrate.

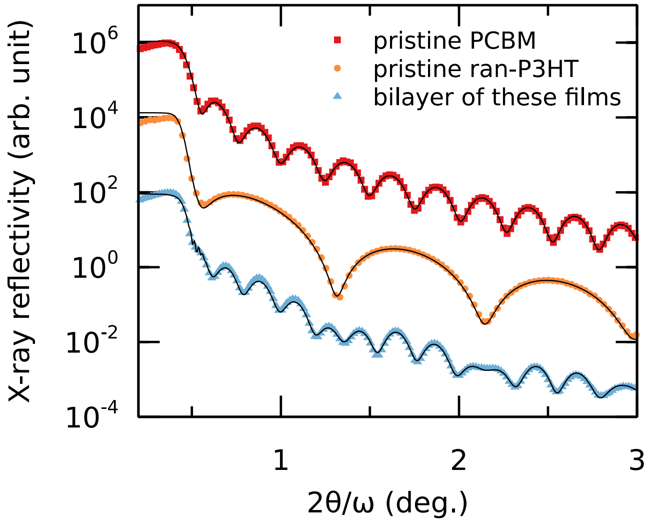

Figure S5. XRR of spin-coated [6,6]-phenyl C_{61} butyric acid methyl ester (PCBM) film after annealing (30 nm) and ran-P3HT films (10 nm) transferred onto the Si/SiO_2 substrate and the bilayer of these two films fabricated by the contact film transfer method. The fittings were performed with a multilayer model (Si/SiO_2/film) with flat interfaces and with the thickness and density of each layer used as the fitting parameters (solid lines). The results of the fitting are summarized below in Table S1. For the bilayer, the curve was well fitted with a double-layer model having a coherent interface and the same density and thickness values of the PCBM and ran-P3HT layers as those before the transfer. Therefore, we concluded that there was no intermixing at the transferred interface.

Table S1. Density and thickness of the layers obtained by fitting the XRR data in Figure S5. Calculated densities agreed with reported values.[1]

Structure	Fitted material	Density (g/cm^3)	Thickness (nm)
Si/SiO_2/PCBM	PCBM (annealed)	1.65	33.6
Si/SiO_2/ran-P3HT	ran-P3HT	1.15	10.4
Si/SiO_2/PCBM/ran-P3HT	PCBM (annealed)	1.68	33.3
	ran-P3HT	1.17	10.4

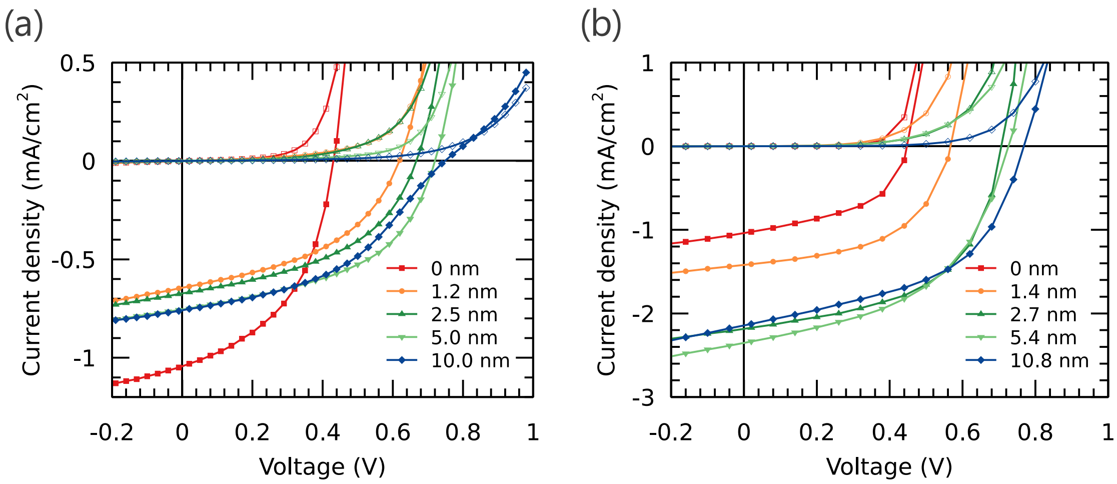

Figure S6. Current density-voltage (*J*-*V*) curves of organic solar cells (OSCs) with (a) PCBM//ran-P3HT//P3HT and (b) PCBM//PTB7//P3HT structures with various interlayer thicknesses and under irradiation of AM1.5 100 mW/cm^2 and in the dark.

Table S2. Device performance of the OSCs with the PCBM//ran-P3HT//P3HT structure and various ran-P3HT interlayer thicknesses. Numbers in parentheses are standard deviations calculated from at least 6 devices.

Thickness (nm)	0	1.3	2.5	5.0	10.0
J_{SC} (mA/cm^2)	1.1 (0.08)	0.67 (0.1)	0.70 (0.05)	0.79 (0.04)	0.79 (0.09)
V_{OC} (V)	0.45 (0.02)	0.60 (0.03)	0.65 (0.03)	0.72 (0.03)	0.74 (0.02)
FF	0.50 (0.04)	0.44 (0.04)	0.44 (0.04)	0.44 (0.04)	0.43 (0.02)
PCE (%)	0.24 (0.03)	0.18 (0.04)	0.20 (0.02)	0.25 (0.02)	0.25 (0.02)

Table S3. Device performance of the OSCs with the PCBM//PTB7//P3HT structure and various PTB7 interlayer thicknesses. Numbers in parentheses are standard deviations calculated from at least 6 devices.

Thickness (nm)	0	1.4	2.7	5.4	10.8
J_{SC} (mA/cm^2)	1.1 (0.08)	1.5 (0.3)	2.2 (0.3)	2.3 (0.2)	2.1 (0.2)
V_{OC} (V)	0.45 (0.02)	0.62 (0.03)	0.74 (0.02)	0.79 (0.04)	0.80 (0.02)
FF	0.50 (0.04)	0.57 (0.05)	0.58 (0.06)	0.52 (0.03)	0.50 (0.02)
PCE (%)	0.24 (0.03)	0.53 (0.1)	0.94 (0.16)	0.96 (0.16)	0.85 (0.13)

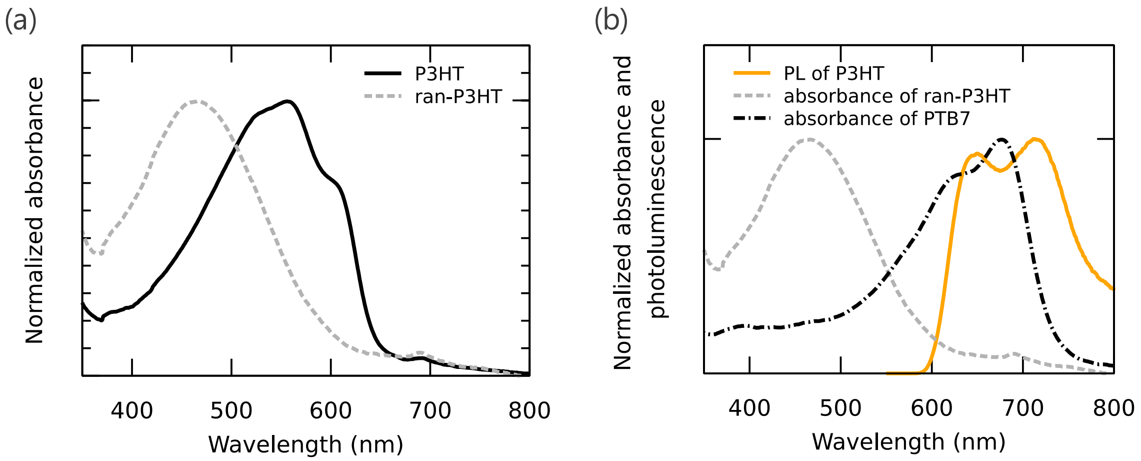

Figure S7. (a) Normalized absorption spectra of P3HT and ran-P3HT thin films. (b) Normalized photoluminescence (PL) spectra of P3HT (λ_{ex} = 450 nm) and normalized absorption spectra of ran-P3HT and PTB7 thin films.

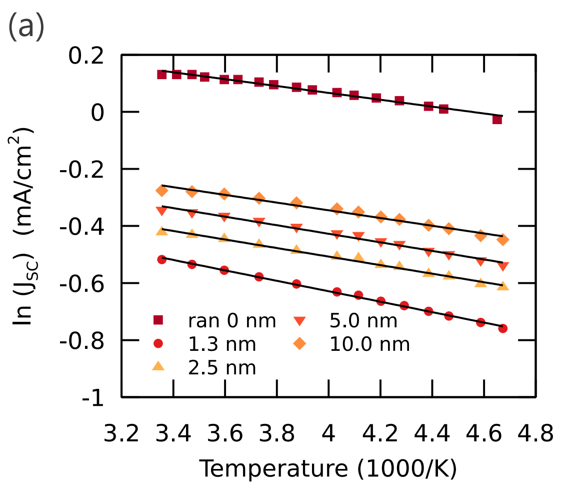

Figure S8. Arrhenius plot of J_{SC} for OSCs with (a) PCBM//ran-P3HT//P3HT and (b) PCBM//PTB7//P3HT structures with various interlayer thicknesses. The activation energy Δ values were derived from the slopes of the lines fit to these data.

Table S4. Activation energy for J_{SC} in OSCs with interlayers with various thicknesses.

Interlayer	ran-P3HT					PTB7				
Thickness (nm)	0	1.3	2.5	5.0	10.0	0	1.4	2.7	5.4	10.8
Δ (meV)	10.4	15.8	12.9	12.9	11.7	10.4	9.3	10.5	20.6	26.4

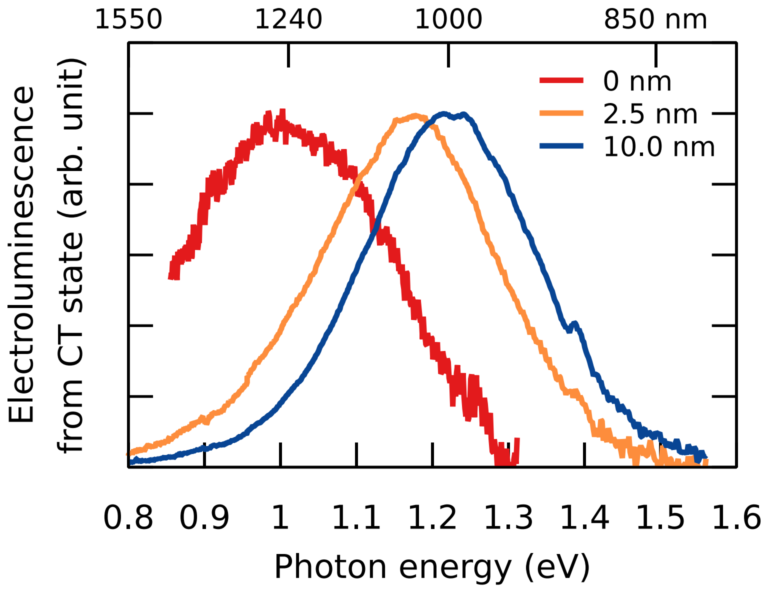

Figure S9. Normalized electroluminescence spectra of OSCs with PCBM//ran-P3HT//P3HT structures and with various thicknesses of the ran-P3HT interlayer (0 nm corresponds to PCBM//P3HT bilayer device).

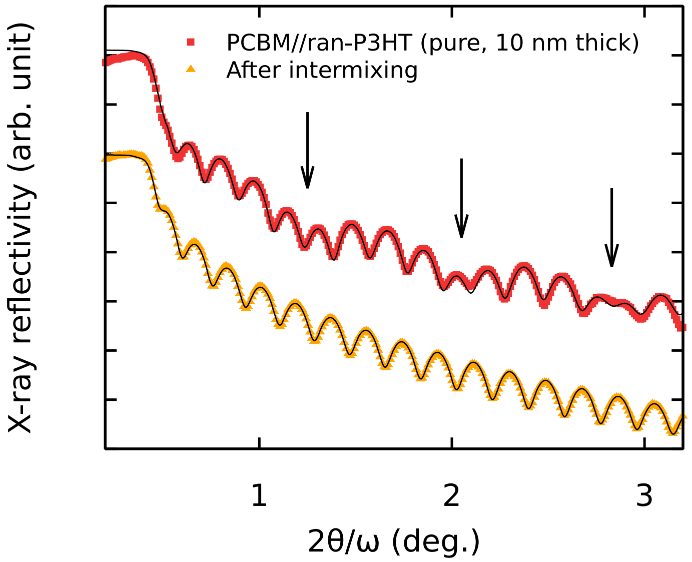

Figure S10. XRR of the PCBM (as-cast)//ran-P3HT bilayer before and after thermal annealing. In the bilayer system, the additional interference pattern from ran-P3HT overlapped with that of PCBM (arrow). In contrast, after intermixing, these overlaps disappeared, meaning that interface between these two layers became obscure.

Table S7. Results of fitting parameters to the XRR data in Fig. S10 for each of the two layers. After the transfer of the ran-P3HT layer (with a thickness of 10 nm) onto PCBM, the data can be fitted using the Si/SiO_2/PCBM/ran-P3HT model with the same density and thickness as before the transfer. After thermal annealing, however, these two layers could not be clearly distinguished from each other, because the obtained density became very similar. Also, the thickness of the PCBM layer slightly decreased, while that of ran-P3HT increased. These changes indicated diffusion of PCBM into the ran-P3HT layer by thermal annealing.

	Before annealing		After annealing	
	PCBM	ran-P3HT	PCBM	ran-P3HT
Density (g/cm^3)	1.54	1.07	1.47	1.51
Thickness (nm)	37.9	10.9	33.8	13.0

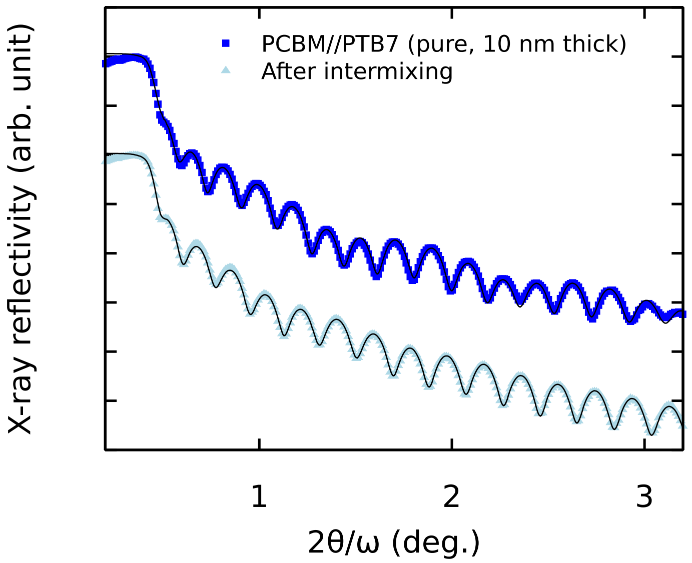

Figure S11. XRR of the PCBM (as-cast)//PTB7 bilayer before and after thermal annealing. Intermixing of PCBM into the interlayer was also observed.

Table S8. Results of fitting parameters to the XRR data in Fig. S11 for each of the two layers. The overall changes in the density and the thickness of the layers here are similar to those in the PCBM//ran-P3HT case, indicating that PCBM also diffused into PTB7 layer by thermal annealing. Note that the PTB7 interlayer after annealing showed a smaller density compared to that of ran-P3HT. This difference might have arisen from the difference of the miscibilities of PCBM in these interlayers. This difference was more clearly observed in the surface analysis by X-ray photoelectron spectroscopy (see the main text and Figure S12).

	Before annealing		After annealing	
	PCBM	PTB7	PCBM	PTB7
Density (g/cm^3)	1.44	1.10	1.53	1.35
Thickness (nm)	37.7	9.6	32.9	12.5

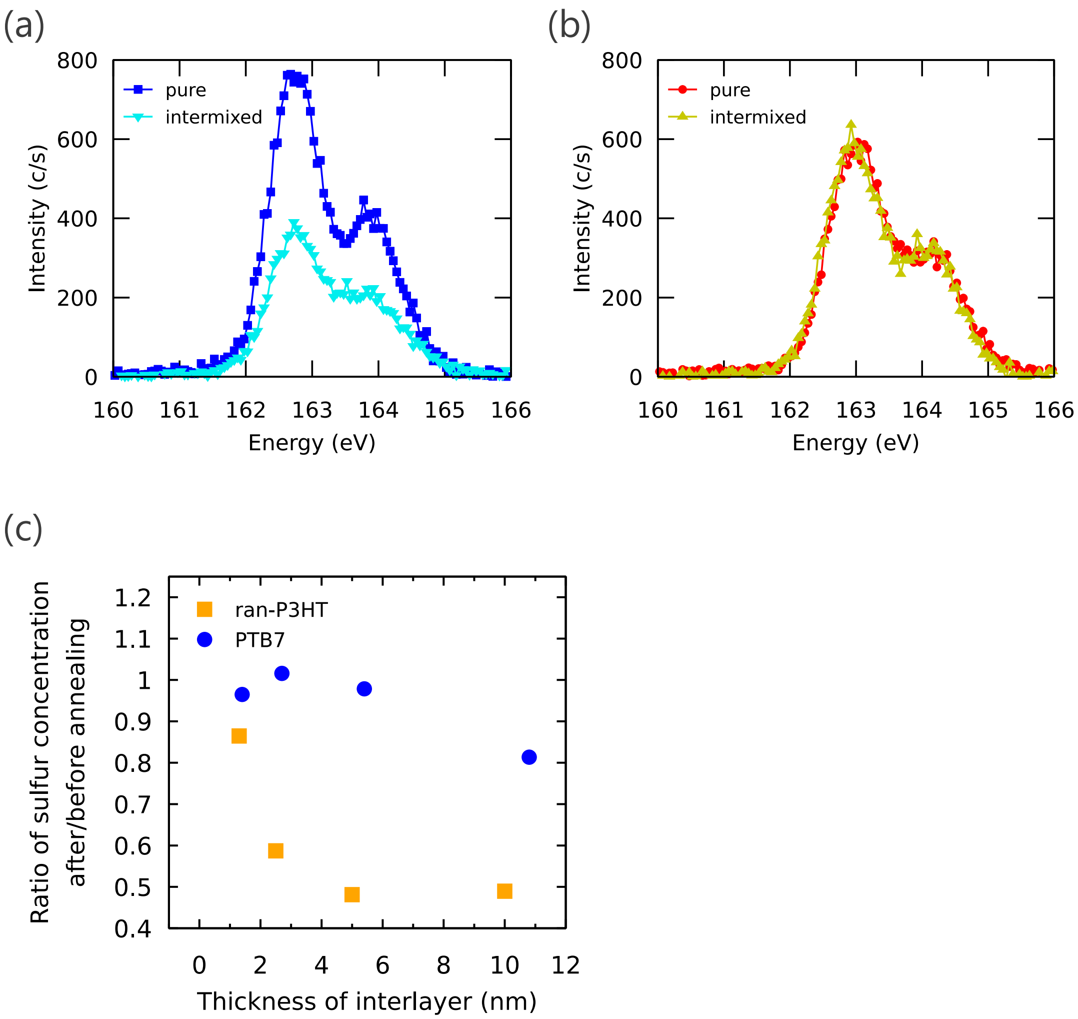

Figure S12. X-ray photoemission spectroscopy (XPS) in the regions of the S2p peak for (a) PCBM//ran-P3HT (5.0 nm) and (b) PCBM//PTB7 (5.4 nm) bilayers before and after thermal annealing. (c) The ratio of the intensity of the S2p peak after annealing to that before annealing plotted against the thickness of the transferred interlayer. The PCBM//ran-P3HT bilayer showed a large decrease in the surface concentration of sulfur after thermal annealing, suggesting that PCBM freely diffused up to the surface of ran-P3HT. In contrast, the PCBM//PTB7 bilayer showed a much smaller decrease in the sulfur concentration on the surface. Therefore, the surface of the PTB7 interlayer contained less PCBM after annealing, probably because of the lower ability of PCBM to diffuse in PTB7.

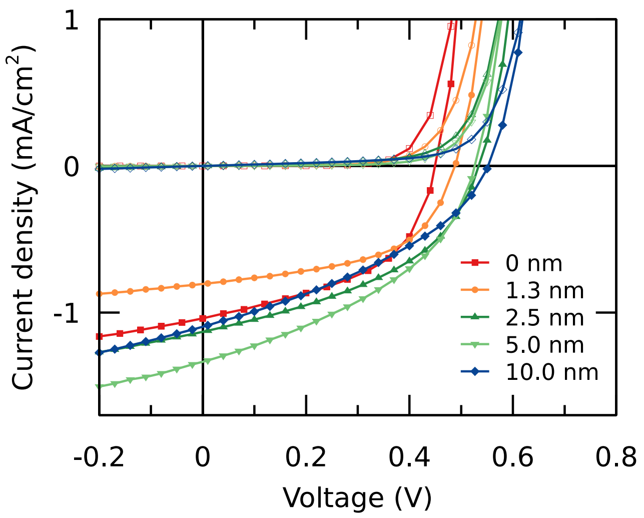

Figure S13. *J-V* curves of the OSCs containing the PCBM/PCBM:ran-P3HT//P3HT structure with various thicknesses of the interlayer under the irradiation of AM1.5 100 mW/cm^2 and in the dark.

Table S9. Device performance of the OSCs with the PCBM/PCBM:ran-P3HT//P3HT structure and various film thicknesses of the mixed interlayer. Numbers in parentheses are standard deviations calculated from at least 6 devices. The last entry is for the PCBM/PCBM:ran-P3HT//ran-P3HT//P3HT structure (see the main text).

Thickness of ran-P3HT (nm)	0	1.3	2.5	5.0	10.0	10.0 (mix)// 1.3 (pure)
J_{SC} (mA/cm^2)	1.1 (0.08)	0.89 (0.1)	1.1 (0.08)	1.3 (0.07)	1.2 (0.04)	1.0 (0.01)
V_{OC} (V)	0.45 (0.02)	0.48 (0.01)	0.51(0.02)	0.54 (0.02)	0.53 (0.03)	0.72 (0.03)
FF	0.50 (0.04)	0.52 (0.02)	0.43 (0.01)	0.41 (0.01)	0.39 (0.02)	0.40 (0.01)
PCE (%)	0.24 (0.03)	0.22 (0.02)	0.25 (0.03)	0.29 (0.03)	0.24 (0.02)	0.29 (0.02)

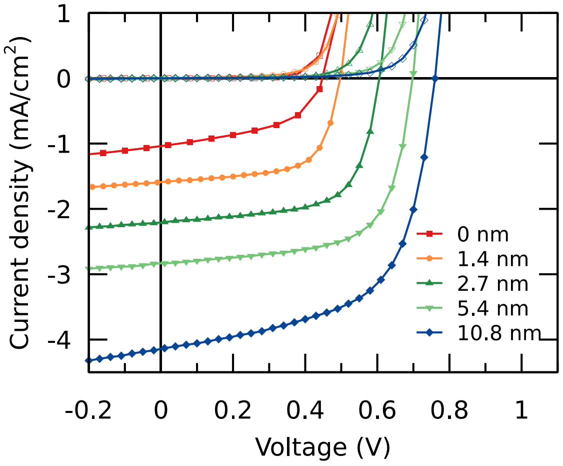

Figure S14. *J-V* curves of the OSCs with the PCBM/PCBM:PTB7//P3HT structure and various thicknesses of the interlayer under the irradiation of AM1.5 100 mW/cm^2 and in the dark.

Table S10. Device performance of the OSCs with the PCBM/PCBM:PTB7//P3HT structure and various film thicknesses of the mixed interlayer. Numbers in parentheses are deviations calculated from at least 6 devices.

Thickness of PTB7 (nm)	0	1.4	2.7	5.4	10.8
J_{SC} (mA/cm^2)	1.1 (0.08)	1.6 (0.17)	2.3 (0.14)	2.9 (0.08)	4.1 (0.13)
V_{OC} (V)	0.45 (0.02)	0.48 (0.02)	0.61 (0.02)	0.69 (0.01)	0.75 (0.01)
FF	0.50 (0.04)	0.65 (0.01)	0.66 (0.01)	0.63 (0.03)	0.59 (0.02)
PCE (%)	0.24 (0.03)	0.50 (0.06)	0.94 (0.08)	1.3 (0.10)	1.8 (0.13)

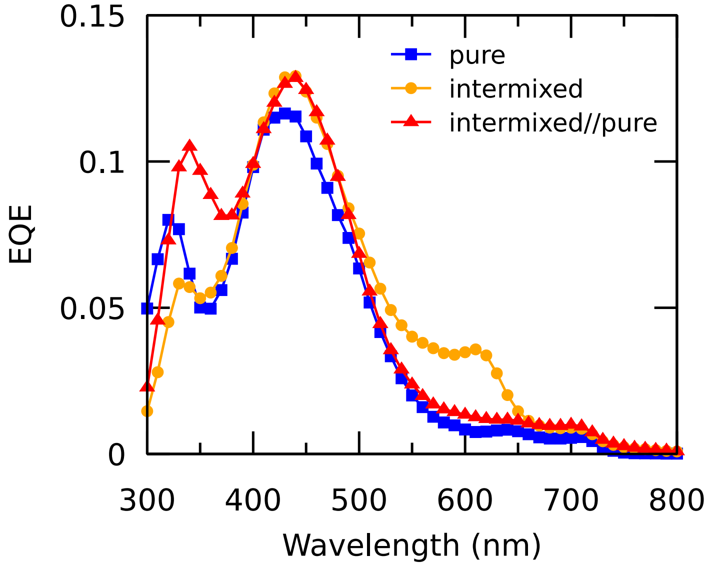

Figure S15. External quantum efficiency (EQE) values of PCBM//ran-P3HT (10 nm)//P3HT heterojunction cells. Pure: no intermixing in the ran-P3HT layer. Intermixed: PCBM was diffused into the ran-P3HT layer. Intermixed//pure: pure ran-P3HT layer (1.3 nm) was transferred onto the intermixed ran-P3HT layer.

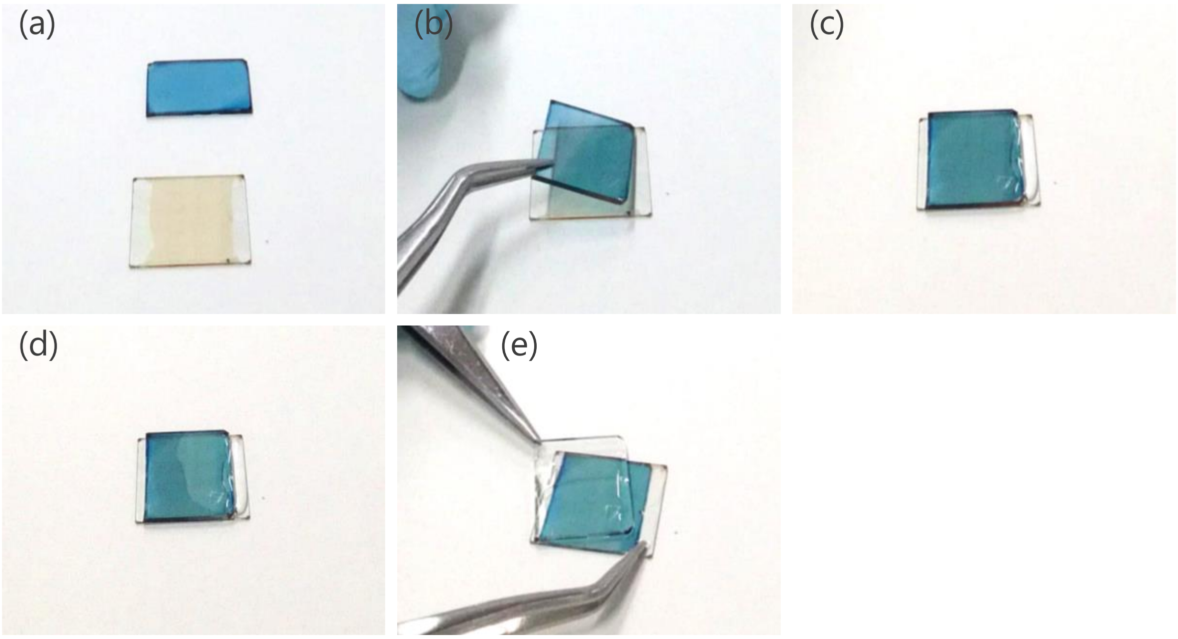

Figure S16. Photographic instruction of each step for the contact film transfer method. (a) The upper substrate is glass/PSS/PTB7 (~40 nm) and the bottom is glass/ITO/ZnO/PCBM (~28 nm). First, (b) the sample of PTB7 is gently placed onto PCBM upside down, then (c) a water droplet is put at the edge of the upper substrate (right side in this case) and (d) the film transfer proceeds from the right to the left. The change of the reflection can be seen as the PSS layer is dissolved by water. After the completion, (e) the glass substrate was removed from the top. The surface of the PTB7 is washed by water for several times to remove the residual PSS.

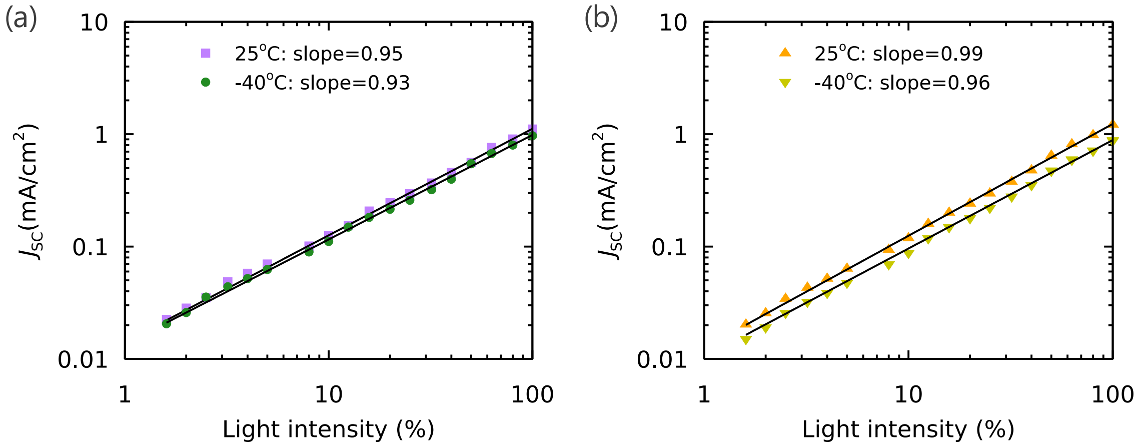

Figure S17. Log-log plots of J_{SC} against the intensity of the irradiated light for the OSCs with (a) PCBM//P3HT bilayer and (b) PCBM//PTB7 (10.8 nm)//P3HT trilayer. An LED light was used as the light source instead of the solar simulator to avoid temperature rising by IR light, thus the absolute values of J_{SC} do not match with those measured under AM1.5, but the energy of LED light at 100% intensity was set to provide almost the same J_{SC}. For both cases, J_{SC} linearly depend on the light intensity in the range of about two orders of magnitude, and their slopes are close to unity even at −40 °C. These results indicate that the bimolecular recombination does not have a large influence on the current generation at the short circuit condition.

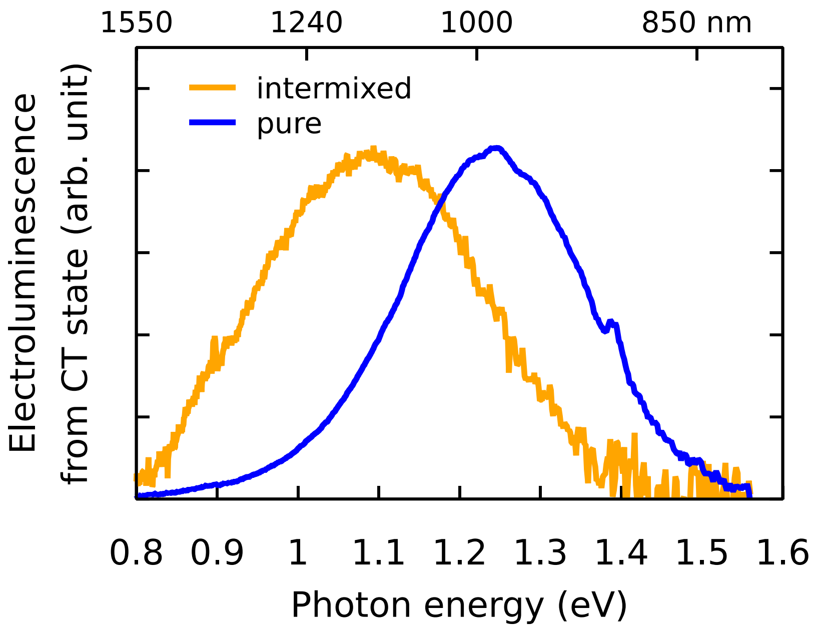

Figure S18. Normalized electroluminescence spectra of OSCs with PCBM//ran-P3HT (7.5 nm)//P3HT structures with and without intermixing in the interlayer.

References

[1] H. W. Ro, B. Akgun, B. T. O'Connor, M. Hammond, R. J. Kline, C. R. Snyder, S. K. Satija, A. L. Ayzner, M. F. Toney, C. L. Soles, D. M. DeLongchamp, *Macromolecules* **2012**, *45*, 6587.
